# The EmpkinS-EKSpression Reappraisal Training Augmented With Kinesthesia in Depression: One-Armed Feasibility Study

**DOI:** 10.2196/65357

**Published:** 2025-04-14

**Authors:** Marie Keinert, Lena Schindler-Gmelch, Lydia Helene Rupp, Misha Sadeghi, Robert Richer, Klara Capito, Bjoern M Eskofier, Matthias Berking

**Affiliations:** 1 Department of Clinical Psychology and Psychotherapy Friedrich-Alexander-Universität Erlangen-Nürnberg (FAU) Erlangen Germany; 2 Machine Learning and Data Analytics Lab Department of Artificial Intelligence in Biomedical Engineering (AIBE) Friedrich-Alexander-Universität Erlangen-Nürnberg (FAU) Erlangen Germany; 3 Translational Digital Health Group Institute of AI for Health Helmholtz Zentrum Muenchen Neuherberg Germany

**Keywords:** depression, cognitive reappraisal, facial expression, kinesthesia, smartphone-based intervention, mobile phone

## Abstract

**Background:**

Harboring dysfunctional depressogenic cognitions contributes to the development and maintenance of depression. A central goal of cognitive behavioral therapy (CBT) for depression is to invalidate such cognitions via cognitive reappraisal (CR). However, relatively low remission rates and high dropout rates in CBT demonstrate the need for further improvement. Potentially, the effects of CBT could be enhanced by addressing not only dysfunctional depressogenic cognitions but also body states associated with depression. This may be done, for example, by systematically pairing the invalidation of depressogenic cognitions with the performance of antidepressive kinesthesia.

**Objective:**

This study aimed to examine the feasibility and clinical potential of a smartphone-based cognitive restructuring task that required users to deliberately perform antidepressive kinesthesia in conjunction with the rejection of depressogenic statements and the affirmation of antidepressive statements. This feasibility study was conducted as a precursor to a large-scale randomized controlled trial.

**Methods:**

In total, 10 healthy participants engaged in a single 90-120-minute session of smartphone-based CR training. During the training, they completed 2 phases in which they were required to reject 20 depressogenic and affirm 20 antidepressive statements, respectively. Diagnostic assessments were conducted 1 week (T1) before and directly prior (T2) to the training, and again directly posttraining (T3) and at a 2-week follow-up posttraining (T4). Feasibility outcomes assessed at T3 included intervention safety recorded by study therapists, compliance, technical feasibility, usability assessed using the Short Version of the User Experience Questionnaire (UEQ-S), and acceptability assessed using the UEQ-S and self-developed items. Preliminary clinical potential was evaluated via single-item ratings of current depressed and positive mood assessed continuously during the training. Feasibility outcomes were analyzed descriptively, and clinical potential was examined using paired-sample *t* tests of pre and post ratings of mood at each training phase.

**Results:**

Overall, the results indicated that the training was safe, feasible, and usable (UEQ-S pragmatic quality scale: mean 1.45, SD 0.71). However, acceptance was limited (UEQ-S hedonic quality scale: mean 1.05, SD 0.79). While 80% (8/10) of the participants were generally satisfied with the training, 80% (8/10) would recommend it to a friend, 90% (9/10) found it interesting, and 80% (8/10) rated it as “leading edge,” 40% (4/10) to 70% (7/10) did not consider it particularly helpful and 50% (5/10) found it repetitive. Preliminary results regarding clinical potential were promising, with significant increases in positive mood (rejection: Hedges *g*=0.63; affirmation: Hedges *g*=0.25), whereas changes in depressed mood were not significant.

**Conclusions:**

This study evaluated the feasibility and acceptability of a smartphone-based CR training augmented with validating and invalidating kinesthesia. This provided valuable insights for further optimizing the intervention for the subsequent randomized controlled trial, but also potential similar interventions. If future studies confirm their clinical potential, such interventions offer a promising approach to enhancing CBT for depression.

**Trial Registration:**

OSF Registries pw6ma; https://osf.io/pw6ma/

## Introduction

Depression is one of the most prevalent mental disorders [1] and severely impacts the quality of life and functioning of affected individuals [2]. Depression is also associated with an increased risk of cardiovascular disease [3] and mortality [4]. Although cognitive behavioral therapy (CBT) has been shown to be effective in depression treatment [5], dropout rates of up to 32 % [6], low remission rates of 42 % [7], and low rates of evidence-based treatment administration [8] highlight the need to further optimize CBT for depression.

CBT for depression is based on Beck’s [9] cognitive model of depression, which assumes that dysfunctional beliefs (eg, “I am worthless”) are a key factor in the etiology and maintenance of the disease. Therefore, one primary goal of CBT is the reappraisal of such dysfunctional beliefs. This is typically pursued with cognitive restructuring techniques, with which therapists guide patients to question the validity of their dysfunctional beliefs and to articulate more adaptive ones [9]. Theory and empirical evidence suggest that invalidating dysfunctional beliefs while strengthening functional beliefs has positive effects on depressed mood and other symptoms of depression [10,11]. To reduce the large treatment gap in depression, researchers have developed accessible and cost-effective CBT-based app interventions that include cognitive restructuring exercises, among others. Such interventions have proven to be effective in reducing symptoms of depression [12]. However, a meta-analysis evaluating single components of such interventions with individual participant data found no clear evidence for the efficacy of cognitive restructuring when used within digital interventions [13], highlighting the need to optimize digital cognitive restructuring interventions. Most of the mobile cognitive restructuring exercises in the context of depression take a similar approach as therapists in face-to-face therapy. They offer psychoeducational content about the relationship between dysfunctional beliefs and depression and provide Socratic questions to help individuals invalidate dysfunctional beliefs and develop more adaptive ones (eg, [14-19]). However, without the support of a therapist, this approach could be very challenging for patients, especially in the context of cognitive and motivational impairments in depression [20,21]. This is also supported by pilot results indicating lower adherence rates with a cognitive restructuring intervention than with an intervention focusing on behavioral activation [18]. Thus, in order to increase adherence, a structured cognitive restructuring exercise with preselected content might be of greater benefit in the context of app interventions without direct human interaction. To our knowledge, only 2 studies evaluate such an intervention. McCloud et al [22] propose a cognitive restructuring exercise based on the ABC technique [23] providing options for alternative interpretations for users to choose from. Stiles-Shields et al [24] propose a step-by-step procedure for identifying and changing maladaptive thoughts using Socratic questions, providing examples of possible thoughts and alternatives. However, the efficacy of McCloud et al [22] cognitive restructuring exercise cannot be determined as it was part of a multicomponent intervention. Stiles-Shields et al [24] conducted a pilot trial. Although they did find effects on symptoms of depression, the informative value is limited due to the small sample size. The efficacy of such structured cognitive restructuring exercises therefore still requires empirical support.

A potential problem of such exercises, particularly if they contain preselected content, could be that the invalidation of dysfunctional beliefs remains superficial and does not lead to emotional insight. This is also assumed by the interacting cognitive subsystems (ICS) theory [25], another seminal theoretical approach in the context of depression. The theory distinguishes intellectual and emotional beliefs and posits that cognitive restructuring only changes dysfunctional beliefs on an intellectual level, but not their “felt meaning.” This is often experienced by patients who report, “I understand the belief is not true, but I still feel that it is.”

ICS suggests that depressogenic thoughts and proprioceptive information interact to generate emotional states in general and depressed mood in particular [25]. Semantic concepts and specific meanings of depressogenic thoughts exist in the form of intellectual beliefs. To generate specific emotional states (eg, depressed mood) and thereby emotional beliefs, however, input from acoustic, visual, and proprioceptive channels (eg, facial expressions, gestures, and posture) is needed. Hence, according to ICS, if patients merely question dysfunctional beliefs on an intellectual level, they will still feel that they are true. To change the resulting emotional state (ie, depressed mood), proprioceptive information needs to additionally contradict the depressogenic schema, for example, assume an upright body posture and confident facial expression. Thus, the maintenance of depression is explained in terms of a coupling of cognitive and proprioceptive feedback loops that sustain each other, referred to as “cognitive interlock.”

In light of these theoretical considerations, we assume that complementing cognitive reappraisal (CR, a key cognitive restructuring technique) with proprioceptions that both invalidate dysfunctional beliefs and validate functional beliefs offers a promising approach to improving CBT for depression. Kinesthesia (ie, proprioception of movement) and, more specifically, facial expressions might be particularly relevant in this context as facial muscles are very sensitive to variations in emotional experience [26].

Empirically, numerous studies provide evidence for a relationship between facial expressions on the one hand, and emotional experience [27] and depression on the other [28-30]. Patients with depression manifest less facial expression of positive affect [28]. Moreover, studies using electromyography (EMG) found greater activity of the corrugator supercilii muscle, a muscle that is activated during negative affective states [31], in response to affective content pictures [32,33], and determined clinical improvement to be associated with attenuated activity of the corrugator supercilii [34]. Consistently, an experimental manipulation of facial expression (ie, inducing a sad facial expression) correlated with negative interpretation bias in healthy adults [35]. The interconnection of depression and facial expression is also reflected in studies using botulinum toxin to block the corrugator supercilii in the treatment of depression, which led to significant and large reductions in symptoms of depression in placebo-controlled studies, as shown by Schulze et al [36] in a recent meta-analysis.

These findings clearly demonstrate the relationship between kinesthesia and depression. However, only very few studies investigated the relationship between kinesthesia and propositional (ie, reasoning) processes in depression. Moreover, to our knowledge, few studies so far have investigated the effects of augmenting cognitive interventions and, more specifically, CR interventions with validating and invalidating kinesthesia. Adaptations of the approach-avoidance modification training (AAMT), which originally required participants to push or pull joysticks to move disorder-relevant images away from or toward themselves [37], use disorder-relevant dysfunctional beliefs as stimuli [38-45]. Two of the studies following such an approach addressed depression (ie, with stimuli such as “I am a failure”), with promising effects on symptoms of depression [38,39]. Moreover, pilot findings from the stress context suggest that using the deliberate display of positive and negative emotions through facial expression, body posture, and a corresponding statement as responses within AAMT might enhance its efficacy [43-45]. Another, albeit not smartphone-based study by O’Toole and Michalak [46] followed an approach more closely aligned with cognitive restructuring in CBT. They evaluated a cognitive restructuring exercise delivered face-to-face combined with emotion-focused body postures and movements and found that it produced greater decreases in agreement with dysfunctional beliefs than isolated cognitive restructuring in healthy individuals.

Although these approaches are promising, evidence remains preliminary, particularly in the context of depression treatment and no study so far has evaluated the efficacy of a smartphone-based structured cognitive restructuring exercise enhanced with validating and invalidating kinesthesia. To advance research in this regard, we developed a structured smartphone-based and sensor-supported CR intervention, in which depressogenic verbalizations are paired with invalidating facial expressions and antidepressive verbalizations are paired with validating facial expressions. Our ultimate goal is to train machine-learning models in the automated assessment of depression, which could then be integrated into a fully automated, smartphone-based biofeedback training. The primary goal of this was to evaluate the feasibility and safety of this intervention in a limited healthy sample by testing its technical setup and procedures before implementation of a large-scale randomized controlled trial (RCT) involving participants with and without depression. The study protocol for the RCT has been published [47]. Second, we aimed to gain preliminary insights into the clinical potential of the intervention, expecting an overall decrease in depressed mood and an increase in positive mood throughout the training and beyond.

## Methods

### Design

In preparation for a large-scale RCT [47], we conducted an experimental feasibility study with a repeated-measures, within-subject design. The study is part of the Collaborative Research Center EmpkinS (Empatho-Kinaesthetic Sensor Technology – Sensor Techniques and Data Analysis Methods for Empatho-Kinaesthetic Modeling and Condition Monitoring) and was conducted at the EmpkinS Lab of the Friedrich-Alexander-Universität Erlangen-Nürnberg (FAU). Participants engaged in a 90-120-minute CR training session augmented with antidepressive facial expression (AFE) prompts on a study-provided smartphone (CR+AFE condition, ie, 1 of the 4 study conditions of the bigger RCT [47]). The study protocol did not involve blinding of either participants or investigators. The study was registered in the Open Science Framework [48] and we have adhered to the American Psychological Association journal article reporting standards [49] when writing the manuscript (Multimedia Appendix 1).

### Participants

Participants were recruited between August 2022 and January 2023. Inclusion criteria were (1) a minimum age of 18 years and (2) sufficient German language skills (at least level B2 according to the Common European Framework of Reference for Languages). Exclusion criteria were (1) a Patient Health Questionnaire (PHQ)-8 score of ≥4, (2) sufficient criteria for any mental disorder (*International Classification of Diseases and Related Health Problems, Tenth Revision* codes F1-F9), (3) acute suicidality, (4) any impairment of facial expression, and (5) dyschromatopsia. We aimed to include N=10 participants, following heuristics from the literature concerning optimal sample sizes for feasibility studies [50,51] and internal considerations of practicality and time constraints. Sociodemographic and clinical baseline characteristics are summarized in Table 1.

**Table 1:** Sociodemographic and clinical baseline characteristics of participants (N=10) in the feasibility study investigating the single-session EmpkinS-EKSpression Reappraisal Training against depression in healthy individuals.

Categories			Values
Age (years; range 18-41), mean (SD)			24.00 (6.31)
Gender: female, n (%)			10 (100)
**Education, n (%)**			
	> 10 years	4 (40)	
	University degree	6 (60)	
**Occupation,** n (%)			
	Student	9 (90)	
	Employed	1 (10)	
Relationship status: single			10 (100)
**Diagnosis of depression, n (%)**			
	Former	1 (10)	
	Current	0 (0)	
**Diagnosis of other mental disorders, n (%)**			
	Former	2 (20)	
	Current	0 (0)	
**Psychotherapeutic or psychiatric treatment**			
	Former, n (%)	4 (40)	
	Current, n (%)	0 (0)	
PHQ-8^a^, mean (SD)			2.10 (0.99)

^a^PHQ: Patient Health Questionnaire.

### Procedures

Participants from the general population were recruited via social media posts targeting the Erlangen area, emails sent via student distribution lists of the FAU, and flyers posted in public places throughout the city of Erlangen. The recruitment material contained information about the conditions for participation, the duration of the study, and the compensation as well as a QR code and link to the study website for registration. The website hosted by Unipark provided information on study aims and procedures. Interested persons were directed to the study screening questionnaire assessing inclusion and exclusion criteria, as well as symptoms of depression with the Patient Health Questionnaire (PHQ)-8 [52] and contact information. Eligible participants were contacted via email or telephone and invited to a diagnostic session (T1). All on-site study sessions were held at the EmpkinS Lab of the FAU. The laboratory rooms were located in a scientific university building and set up specifically for this study. At the beginning of the diagnostic session participants provided written informed consent. We then conducted the Structured Clinical Interview for DSM-5 Disorders-Clinician version (SCID-5-CV; German version: [53]) to assess the diagnostic status of participants. After the interview, participants answered a set of questionnaires via Unipark.com (T1).

One week after the diagnostic session, the training session was held at the Lab. The entire session took about 3 hours. First, we conducted the GRID Hamilton Depression Rating Scale (GRID-HAMD; [54]) to assess depression. Next, participants answered a web-based preassessment (T2), also via Unipark.com and the physiological data acquisition was set up. Then, participants received a short psychoeducation explaining the interconnection of thoughts, mood, and the body. The subsequent smartphone-based training session took approximately 90-120 minutes. After completing the training, a short postassessment (T3) was completed, again via Unipark.com.

Two weeks after the training session, participants were invited to a follow-up assessment (T4). The GRID-HAMD was conducted via telephone and participants were asked to fill out an online questionnaire via Unipark.com. They received the link to the questionnaires via email one day before the telephone interview.

The interviews and study sessions were conducted by trained clinical psychologists and based on manuals outlining study procedures and instructions for participants.

### Smartphone Intervention

**Figure 1:**
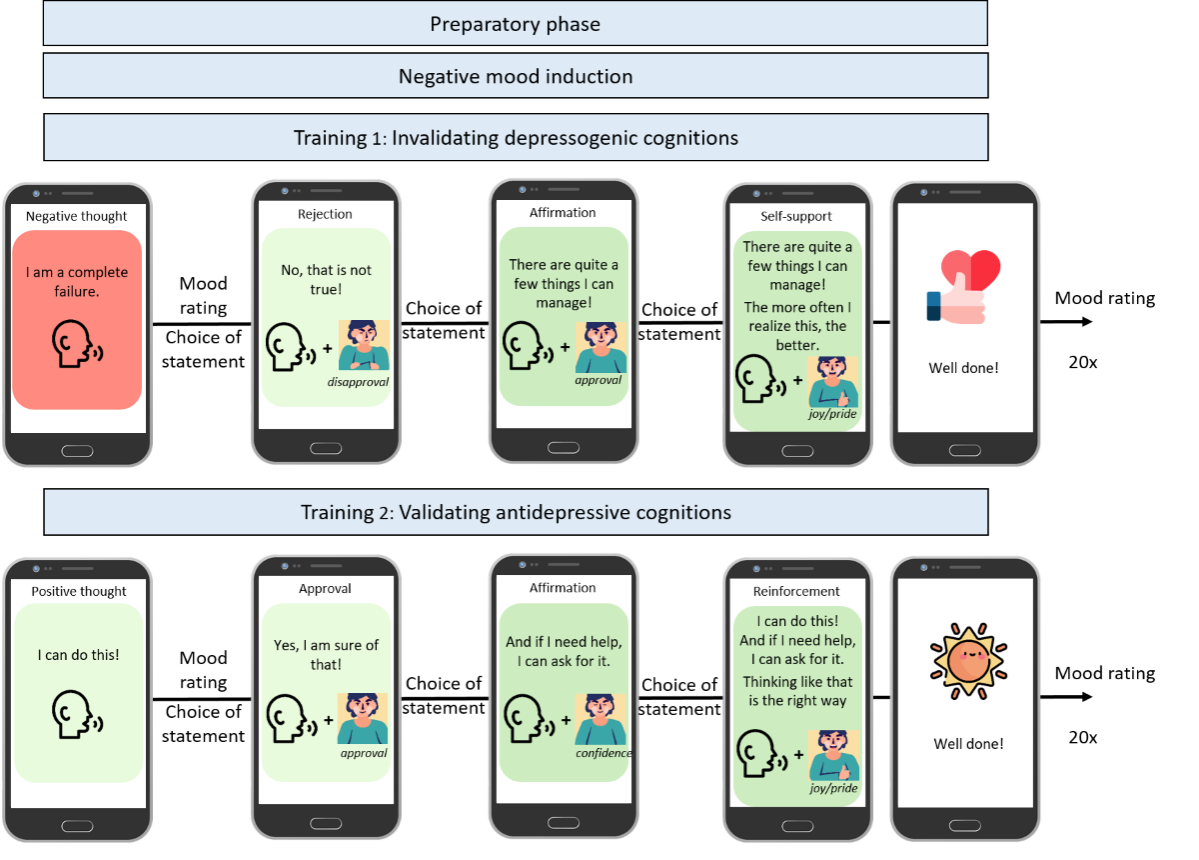
Intervention procedure of the single-session EmpkinS-EKSpression Reappraisal Training against depression was examined in this feasibility study in healthy individuals.

During the training, participants completed four phases (Figure 1): (1) the preparatory phase, (2) the depressed mood induction phase, (3) the first training phase, and (4) the second training phase. During the preparatory phase, participants were shown a fixation cross (3 sec) followed by different stimuli (8 sec) in randomized order on the smartphone. The stimuli consisted of self-recordings of the participants, words congruent and incongruent with depressed mood (ie, “hopeless” and “happy”), and words representing the so-called cognitive triad of depression (ie, “me,” “the world,” and “the future” [55]), which were each shown 4 times, resulting in 24 stimuli in total. Subsequently, in order to allow working with current depressed mood during the training, participants received a validated mood induction beforehand. During this mood induction phase, participants read aloud ten depressogenic cognitions shown on the smartphone screen (eg, “My future is absolutely hopeless”). In addition, sadness-inducing music was played (an excerpt from “Adagio in G minor” by Tomaso Giovanni Albinoni). The induction procedure was previously validated [56,57].

Both training phases started with the study therapist providing instructions on how to engage in the training, followed by on-screen instructions with text and animations in the EmpkinS-EKSpression app. Then, participants completed 2 practice trials before starting the actual training. In the actual training, participants were confronted with 20 depressogenic statements (eg, “I am a complete failure”; first training phase) and antidepressive (eg, “I can do this;” second training phase) statements presented in randomized order on the smartphone screen. The statements were derived from validated questionnaires in the context of depression (Beck Hopelessness Scale, German version [58]; Cognitive Triad Inventory, German version [59]; Automatic Thoughts Questionnaire, German version [60]; and Rosenberg Self-Esteem Scale, German version [61]). A team of 3 experts in clinical psychology adapted the items to customize them for the intervention.

In the first training phase, participants were asked to reject the depressogenic statements in the following three invalidation steps: (1) reject the presented cognition, (2) affirm the rejection, and (3) self-support themselves. In each step, participants were able to choose from three statements (ie, 3 statements expressing rejection, affirmation, and self-support, respectively) which they had to manually select on the screen and also read aloud. Their verbalization was to be amplified by the explicit expression of congruent kinesthesia, including mimic, gesture, and posture. Thus, their rejection of each depressogenic statement was performed with an expression of disapproval, the affirmation with an expression of approval, and self-support with an expression of joy (trials 1-10) and pride (trials 11-20).

In the second training phase, participants received instructions to validate the antidepressive statements in the following sequence: (1) approve the positive cognition with an expression of approval, (2) affirm the approval with an expression of confidence, and (3) reinforce themselves with an expression of joy (trials 1-10) and pride (trials 11-20). Here, to conclude, participants read aloud all 3 statements they had selected.

After each trial, reinforcing statements and pictures were displayed on the screen. Throughout the sequence of training trials, study therapists rated the persuasiveness of participants’ emotional expression, provided feedback to participants, and maintained control of the training app using a tablet.

### Measures

#### Feasibility of the Intervention

To determine the feasibility of the intervention, we assessed safety, technical feasibility, compliance, usability, and acceptability.

##### Safety of the Intervention

To determine the safety of the intervention, study therapists recorded whether participants experienced significant mood deterioration during the training. The intervention was deemed safe if no more than 10% (1/10) of the training sessions were prematurely terminated due to significant mood deterioration.

##### Compliance

The compliance rate was defined as the number of participants completing the intervention per protocol. The intervention was considered feasible if the compliance rate was at least 50% (5/10).

##### Technical Feasibility

Technical difficulties with the EmpkinS-EKSpression app during the training were to be reported by study therapists. We considered the intervention feasible if at least 80% (8/10) of the sessions were completed without major technical problems.

##### Usability

The usability of the training app was assessed with the pragmatic quality scale of the short version of the User Experience Questionnaire (UEQ-S [62]) and with one self-constructed item. In the UEQ-S, 8 pairs of opposite adjectives related to the user experience are rated on a 7-point Likert scale from –3 (fully agree with the negative term) to +3 (fully agree with the positive term). The pragmatic quality scale consists of 4 of the 8 items, with the other 4 items belonging to the hedonic quality scale. The self-constructed item asked participants how intelligible the app instructions were on a 4-point Likert scale from 1 (not at all) to 4 (very). We considered the intervention usable if >80% (8/10) of participants rated the app as positive (score ≥1), that is, supportive (rather than obstructive), easy (rather than complicated), efficient (rather than inefficient), clear (rather than confusing), and very intelligible.

#### Acceptability

Acceptability of the intervention was assessed with the “hedonic quality” scale of the UEQ-S. The intervention was considered acceptable if >80% of participants rated the training as positive (score ≥1) that is, interesting (vs not interesting), exciting (vs boring), inventive (vs conventional), and leading edge (vs usual). A total of 12 additional self-generated items were used to assess acceptability (Multimedia Appendix 2 provides the items). Except for 2 items, they were all rated on a 4-point Likert scale from 1 (not at all) to 4 (very). One item asking whether participants would recommend the training to a friend was answered with yes or no. Another item asking whether the training takes a reasonable amount of time was rated on a 5-point Likert scale from 1 (far too high) to 5 (far too low). The intervention was considered acceptable if >80% of participants indicated they would recommend the training to a friend, were rather content with the training, would continue the training given the opportunity, considered the training as rather helpful, and fun (score ≥3), as well as requiring a reasonably acceptable amount of time (score 2-4), and rate the training as only a little or not at all strenuous and difficult to focus on (score ≤2). Moreover, open-ended questions were asked by study therapists during the training and at the T3 assessment to gain additional, qualitative feedback for improvement. Acceptability of the overall study was assessed with one item asking participants to rate how burdensome study participation had been on a 4-point Likert scale from 1 (not at all) to 4 (very). The study was considered acceptable if >80% of participants rated participation as only a little or not at all burdensome.

#### Clinical Measures

The primary clinical outcome was current depressed mood and the secondary outcome was current positive mood.

Current depressed mood was assessed using an 11-point Likert scale from 0 (no depressed mood at all) to 10 (very strong depressed mood) during both diagnostic and training sessions. During training, participants submitted 108 ratings in total: 24 during the preparatory phase, one after the negative mood induction, one immediately before the first training item, one after each initial statement, and one after the completion of each training trial.

Current positive mood was also assessed using an 11-point Likert-scale from 0 (no positive mood at all) to 10 (very strong positive mood). Similar to the number of ratings of depressed mood, participants submitted a total of 108 ratings of positive mood.

#### Demographic and Health-Related Information

Demographic and health-related variables included age, date of birth, German language skills, nationality, gender, height, weight, academic degree, professional occupation, relationship status, scholarly fields of study (if any), current or previous diagnosis of a mental illness, psychotherapy, medication, color blindness, and Botox treatment. In addition, the PHQ-8 [52] was used to assess current depressive symptom severity during diagnostic screening. The PHQ-8 consists of 8 items assessing the frequency of 8 of the 9 *Diagnostic and Statistical Manual of Mental Disorders* criteria of depression during the previous 2 weeks on a 4-point Likert scale (0=not at all to 3=nearly every day). The items are added up to a sum maximum score of 24, with a score ≥10 indicating clinically relevant symptom severity [52].

#### Additional Measures

As one aim of our feasibility study was to test the technical setup of the study and protocol procedures, we included all measures to be assessed in the RCT. However, the additional measures listed below will not be analyzed in this feasibility evaluation. The clinical status of participants was assessed with the SCID-5-CV during diagnostic screening. Symptoms of depression were assessed with the GRID-HAMD [54] during the training session and at follow-up. In addition, self-reported symptoms of depression were assessed with the Centre for Epidemiological Studies Depression Scale (German version [63]). Current suicidality was assessed with a single self-developed item. Dysfunctional attitudes were assessed with the German short version of the Dysfunctional Attitude Scale, form A [64], and automatic thoughts with the German version of the Automatic Thoughts Questionnaire-Revised [60]. Finally, the emotional state was assessed with the Self-Report Instrument for the Assessment of Emotion-Specific Regulation Skills parts A and B (SEK-ES [65]).

Two video cameras were used to assess kinesthesia (ie, facial expression and body posture): an Azure Kinect depth camera (Microsoft) and a high-resolution RGB camera (Sony SRG-300H), positioned 1.2 meters in front of participants. In addition, bipolar EMG of various facial muscles (ie, corrugator supercilii, zygomaticus major, masseter, orbicularis oculi) and of the shoulder muscle (trapezius) were assessed with the BioPac MP160 system (Biopac Inc). Further psychophysiological measures assessed with the BioPac MP160 system included electrocardiogram, respiration (using a respiration belt), and electrodermal activity. In addition, heart rate and respiration were assessed with radar-based technology [66]. The preparatory phase served as a baseline period for physiological data acquisition. Further, we assessed pupillometry data. Moreover, the smartphone app recorded videos of each trial and participants’ digital activity on the app using the smartphone’s front camera.

### Statistical Analysis Plan

The safety and feasibility of the intervention and collection of kinesthetic and physiological data were determined based on descriptive analyses. Results were compared to predefined threshold values (see above). As a manipulation check, we analyzed whether the negative mood induction successfully elicited an increase in depressed mood and a decrease in positive mood. To this end, a paired-sample *t* test was computed to compare depressed or positive moods before and after the negative mood induction. To analyze the clinical potential of the intervention, we also computed paired-sample *t* tests to compare the mean rating of depressed (dependent variable 1) and positive mood (dependent variable 2) after the presentation of the statements, with the mean depressed and positive mood rating after completion of the trials. We analyzed the clinical potential separately for the 2 training phases. For effect sizes, we computed Hedges *g* as proposed by Lakens [67] for small samples, with Hedges *g* values of 0.2, 0.5, and 0.8 representing small, moderate, or large effects, respectively [68]. Moreover, we implemented 2 linear mixed-effects models with time-point as a fixed effect on level 1 and random intercepts and random slopes to analyze the course of depressed and positive mood ratings over the course of the training. The dependent variables were depressed mood and positive mood, respectively. However, as our data showed too little variance, particularly during the second training phase, the models appeared to not converge. Therefore, we merely analyzed the course of depressed and positive mood on a descriptive level.

The level of significance for all analyses was α=.05. The analyses were conducted with R Studio (version 4.3.2; Posit) [69].

### Ethical Considerations

The study followed the Declaration of Helsinki’s ethical guidelines and obtained ethical approval from FAU’s Ethics Committee (20-443-B, 20-443_1-B, 20-443_2-B). All participants provided written informed consent at 2 time points during the study: (1) for the screening at the beginning of the online questionnaire via Unipark and (2) for the rest of the study at the beginning of T1. To deidentify questionnaire and app data, each participant received a personal code. A list connecting personal information with the code was stored on a secure server and accessible by study staff only. Video and audio data collected during the study that could not be completely anonymized were stored on an external hard drive that was locked away at all times.

Participants received compensation up to €40 (€10 for the diagnostic session, €20 for the training session, €5 for the follow-up assessment, and a €5 bonus for completing the whole study; a currency exchange rate of 1€=US $1.09 is applicable). Psychology students at FAU could alternatively receive course credits.

## Results

### Feasibility of the Intervention

**Figure 2:**
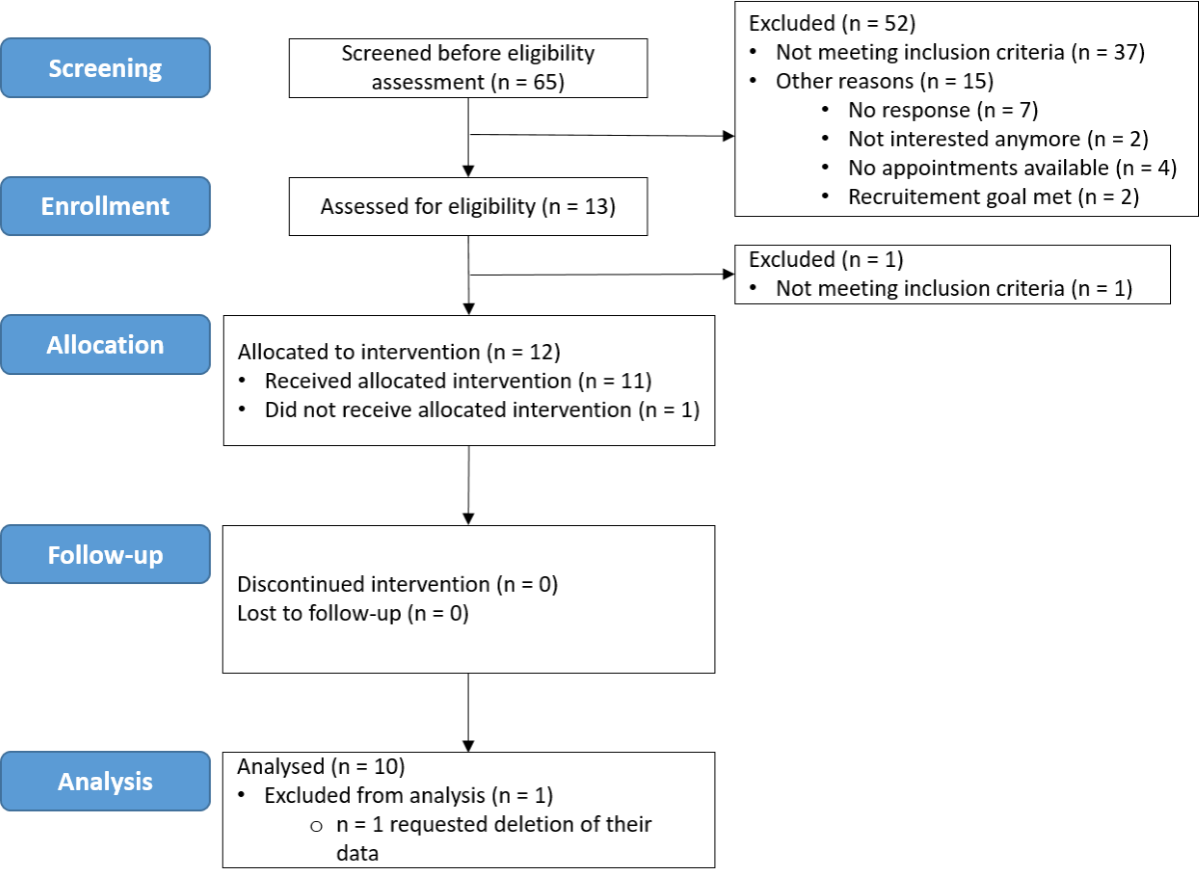
Consolidated Standards of Reporting Trials (CONSORT) flow diagram of participants in the feasibility study investigating the single-session EmpkinS-EKSpression Reappraisal Training against depression in healthy individuals from screening to analysis.

The study flow is displayed in Figure 2. Regarding safety and compliance, none of the participants terminated the training prematurely. Technical difficulties with the EmpkinS-EKSpression app occurred in 2 training sessions: one of them had to be terminated immediately after the start due to an error in the app, leading to the drop-out of that participant (Figure 2). The other session was terminated after the first training phase also due to an error in the app. Here, the data recorded by the smartphone app (including mood ratings delivered throughout the training) were missing. Therefore, this participant was not included in the analysis of clinical potential. However, the data were included in the feasibility analyses.

With regards to usability as assessed with the pragmatic quality scale of the UEQ-S, favorable results emerged. Except for the instrument’s “complicated – easy” item, all items were rated positively (Table 2). The total score of the pragmatic quality scale was a mean of 1.45 (SD 0.71). A total of 70% (7/10) of participants assessed the overall usability positively. Ratings for our self-developed item on the intelligibility of the app instructions ranged from 3 to 4 with a median of 4 (mean 3.9, SD 0.32). 90% (9/10) of participants rated the instructions as very intelligible.

**Table 2:** Descriptive statistics of the short version of the User Experience Questionnaire assessing the usability of the single-session EmpkinS-EKSpression Reappraisal Training against depression examined in this feasibility study in healthy individuals.

			Median score		Score range		Scores, mean (SD)		Participants (N=10) with scores meeting benchmark^a^, n (%)
**Pragmatic quality scale**									
	Obstructive–supportive	2		0 to 3		1.8 (0.79)		9 (90)	
	Complicated–easy	2		–3 to 3		1 (2.11)		6 (60)	
	Inefficient–efficient	1		–1 to 2		1.1 (0.99)		8 (80)	
	Confusing–clear	2		–1 to 3		1.9 (1.29)		9 (90)	
**Hedonic quality scale**									
	Not interesting–interesting	1.5		0 to 3		1.5 (0.85)		9 (90)	
	Boring–exciting	0.5		–1 to 2		0.2 (1.14)		5 (50)	
	Conventional–inventive	1.5		–2 to 3		1.1 (1.45)		7 (70)	
	Usual–leading edge	2		–2 to 3		1.4 (1.51)		8 (80)	

^a^The predefined benchmark for the items considered to be rated as positive was a score ≥1. The percentage of participants meeting this benchmark is displayed here.

For acceptability, as assessed with the hedonic quality scale of the UEQ-S, results were more mixed (Table 2). While the total score was a mean of 1.05 (SD 0.79), only 50% (5/10) of participants gave a positive total score for the hedonic quality scale. Regarding our self-developed items, 80% (8/10) of participants indicated they would recommend the training to a friend. Further descriptive statistics are displayed in Table 3. In the open evaluation of the training, 4 participants revealed that they found the training repetitive; 2 found it exhausting. A total of 5 participants found the emotional display, 3 pride in particular, initially unfamiliar and difficult to engage in. Moreover, 5 participants were critical that the standardized statements were not better tailored for individuals, and 3 were dissatisfied with the study setting (ie, uncomfortable seating position, feeling observed by the study therapist, and awkward electrodes). In contrast, some positive feedback included that the training was fun and interesting (n=4), that it provided new ideas for dealing with negative thoughts (n=4), and that there were several statements to choose from (n=1). Regarding the acceptability of the study, participation burden ratings ranged from 1 to 2, with a median of 1 (mean 1.1, SD 0.2), hence, 100% (10/10) of participants rated the participation as only a little or not at all burdensome.

**Table 3:** Descriptive statistics of self-constructed items assessing the acceptability of the single-session EmpkinS-EKSpression Reappraisal Training against depression examined in this feasibility study in healthy individuals (N=10).

		Median score			Score range	Scores, mean (SD)	Participants (N=10) with scores meeting benchmark^a^, n (%)
Likelihood of continuation		2			1 to 3	1.9 (0.74)	2 (20)
Satisfaction in general		3			2 to 4	2.9 (0.57)	8 (80)
**Helpfulness**							
	Training part 1		2	1 to 4		2.4 (0.84)	4 (40)
	Training part 2^b^		3	2 to 3		2.67 (0.5)	6 (60)
	Training in total		3	2 to 3		2.6 (0.52)	6 (60)
	Modifying negative thoughts		2	2 to 3		2.3 (0.48)	3 (30)
	Coping with negative thoughts		2	2 to 4		2.5 (0.71)	4 (40)
	Enjoyment		2	2 to 3		2.4 (0.52)	4 (40)
	Strenuousness		2	2 to 3		2.2 (0.42)	8 (80)
	Reasonable amount of time		2	2 to 4		2.4 (0.7)	3 (30)
	Difficulty to focus		1	1 to 2		1.3 (0.48)	10 (100)

^a^The predefined benchmark for the items considered to be rated as positive was a score ≥3 for the likelihood of continuation, satisfaction in general, helpfulness, and enjoyment; a score between 2 and 4 for a reasonable amount of time; and a score≤ 2 for strenuousness and difficulty to focus.

^b^N=9.

### Manipulation Check

With regards to negative mood induction, the mean rating of depressed mood was 0.5 (SD 1.41) before and 1.44 (SD 0.73) after mood induction. The mean rating of positive mood was 5.67 (SD 1.66) before and 4.56 (SD 1.88) after negative mood induction. Both paired-samples *t* tests reached significance (*t*_7_=–3.06, *P*=.02, Hedges *g*=–0.69, 95% CI –2.83 to –0.28 and *t*_8_=3.16, *P*=.01, Hedges *g*=–0.95, 95% CI –1.84 to –0.55; respectively).

### Clinical Potential

**Figure 3:**
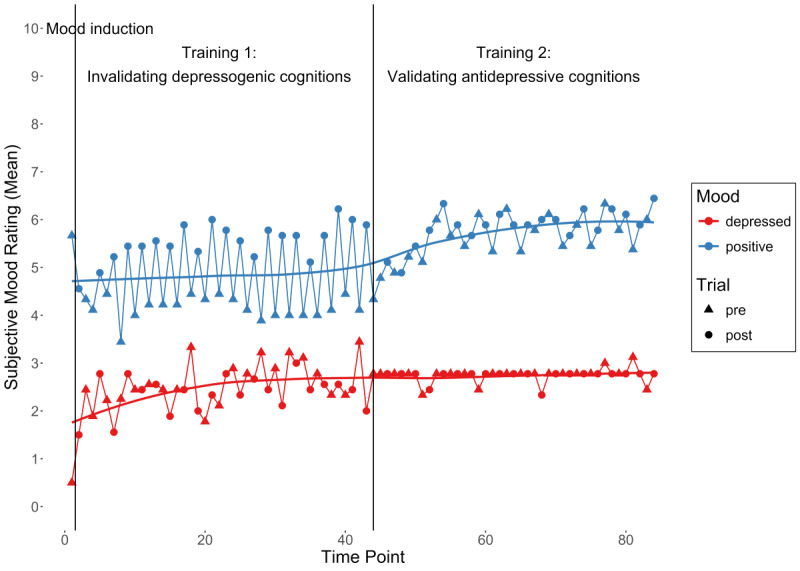
Subjective depressed and positive mood ratings over the course of the single-session EmpkinS-EKSpression Reappraisal Training against depression were examined in this feasibility study in healthy individuals. Regression lines represent loess curves.

In the first training phase, the mean rating for depressed mood was 2.63 (SD 2.51) before and 2.41 (SD 2.36) after, and the mean rating for positive mood was 4.14 (SD 2.42) before and 5.59 (SD 1.65) after the explicit rejection of depressogenic statements, respectively. The difference was not significant for depressed mood (*t*_178_=1.16, *P*=.25, Hedges *g*=0.09, 95% CI –0.07 to 0.23), but did reach significance for positive mood (*t*_179_=–8.53, *P*<.001, Hedges *g*=–0.63, 95% CI –0.72 to –0.55). In the second training phase, the mean rating of depressed mood was 2.75 (SD 2.47) before and 2.74 (SD 2.48) after, and the mean rating of positive mood was 5.62 (SD 1.71) before and 5.81 (SD 1.58) after the explicit substantiation of antidepressive statements, respectively. The difference was not significant for depressed mood (*t*_178_=–0.16, *P*=.87, Hedges *g*=–0.01, 95% CI –0.16 to 0.12), but did, again, reach significance for positive mood (*t*_177_=–3.38, *P*<.001, Hedges *g*=–0.25, 95% CI –0.38 to –0.11]). Descriptively, there was a visible mood change after the negative mood induction (ie, an increase in depressed mood, and a decrease in positive mood) and an increase in positive mood after each trial, particularly during the first training phase (Figure 3). Moreover, there was a slight increase in depressed mood over the course of the first training phase, and in positive mood over the course of the second training phase.

## Discussion

### Principal Findings

This study aimed to assess the safety, feasibility, and clinical potential of the novel EmpkinS-EKSpression training that leverages the power of AFE to augment the beneficial effects of CR on depression. Overall, the intervention proved to be safe and feasible, and findings on its clinical potential were promising. However, we identified several aspects that warrant further clinical and technical optimization.

With regard to feasibility, the results were quite positive, and the predefined benchmarks were met for safety, compliance, and technical feasibility. Concerning the usability of the training app as assessed with the pragmatic quality scale of the UEQ-S and a self-developed item on the intelligibility of the app instructions, the predefined benchmark was narrowly missed. This result is worse than the findings of Stiles-Shields et al [24] who evaluated a structured cognitive restructuring exercise where usability was rated to be high. An important, possibly explanatory difference in this study is a higher degree of individualization: participants could choose between self-generated and preselected content in each step of the cognitive restructuring task. It is possible that including more options for individualization of content might improve the usability of the EmpkinS-EKSpression training. Similarly, one of the studies investigating an AAMT intervention in the context of depression reported high usability ratings [39]. The training content here was highly standardized, so a lack of individualization might not be the only explanatory factor for missing the usability benchmark in this study. One difference was the amount of time spent on the cognitive restructuring task (which was not reported by Stiles-Shields and colleagues [24]). While individuals in our study trained for 90-120 minutes, individuals in the study by Lukas and Berking [39] completed an average of around 40 minutes of training. Therefore, a shorter treatment duration might be beneficial to increase usability. When analyzing our usability results at an item level, only the results for the “complicated – easy” item did not meet the benchmark in our study. The variance of the item emerged to be high, and the median indicates that most participants rated the app as easy. Thus, the fact that the benchmark was missed here is likely attributable to singular, extreme votes. Nevertheless, the explicit display of antidepressive kinesthesia might be more challenging than mere cognitive restructuring exercises. Therefore, this finding should be considered in future studies using similar paradigms with rather complicated setups, such as AAMT, to emphasize a user-friendly development of interventions and involve target groups to address their needs, for example, by improving study support or providing more information material. We decided to address this issue in the upcoming RCT by providing a more in-depth oral explanation by study therapists during the training phase. Results on the acceptability of the training, as assessed with the hedonic quality of the UEQ-S, as well as several self-constructed items, did not meet the predefined benchmarks. The open evaluation revealed that over half of the participants found the training to be too repetitive and too long. This finding is in line with previous work investigating the acceptability of a digital cognitive bias modification training that also required participants to complete many trials, which suggests that participants’ willingness to engage in repetitive digital treatments is limited [70]. However, from the ICS theoretical perspective, repeated rehearsal of new and adaptive schemas is important to break the cognitive interlock and thus the maintenance of depressed mood [25]. One possible solution to this dilemma, as suggested by Beard and colleagues [70], is to increase the credibility of the intervention by ensuring a highly comprehensible and convincing treatment rationale and motivation. This suggestion is also in line with a systematic review of barriers and facilitators for digital mental health interventions, which identified credible content of an intervention to be an important facilitator for user engagement [71]. Our feasibility study was likely to fall short of meeting this requirement, given that we only included healthy individuals with perhaps only a limited need for a mood enhancement intervention. Thus, the content of the training was probably not sufficiently relevant for participants, which in turn could have compromised its credibility. However, it is common practice for feasibility studies to initially scrutinize general intervention effects in healthy individuals before exposing more vulnerable clinical participants to those effects [72]. Nevertheless, we took this as an indication to expand our study instructions and elaborate on the study rationale for the subsequent RCT. This approach may also be useful to increase the face validity and thus potentially the effectiveness [73] of other digital mental health interventions, such as structured cognitive restructuring exercises [22,24], smartphone-based AAMT used for CR [38-45], or original AAMT versions used to retrain approach and avoidance biases (Loijen et al performed a systematic review [74]).

The fact that our feasibility sample consisted of healthy individuals might also explain the finding that participants did not consider the training to be particularly helpful. If they did not relate to the standardized depressogenic statements in the first place (which was reflected in participants’ open feedback), then there were no negative underlying cognitions to be modified. In terms of strain associated with study participation and training, the benchmark was met for all items. However, 7 participants indicated that the training session was too protracted. Therefore, we decided to abbreviate the preparatory phase of the training to the presentation of only 12 instead of 24 stimuli for 5 instead of 8 seconds. The primary purpose of the preparatory phase was to gather baseline physiological data, which was shown in the feasibility study to be adequately achieved in a shorter time frame. We also decided to move the GRID-HAMD interview to the diagnostic session (T1) to reduce participant burden during the subsequent training session. Another important feedback for the training was that participants expressed difficulty in fully engaging in the spontaneous display of emotion. As a result, the RCT will incorporate increased support to participants from study therapists during the training. One implication for future interventions using antidepressive kinesthesia to augment cognitive restructuring derived from this feedback is that such interventions should be therapist-supported, at least when they are first delivered. Such support can maximize the intervention’s effects by leveraging the therapeutic relationship, which is a crucial common factor of psychotherapy [73]. This is also consistent with studies demonstrating that therapist-supported digital interventions for depression are more effective than self-directed interventions [75]. Furthermore, one participant reported that they found the electrodes uncomfortable, which was also witnessed by study therapists. Therefore, for the subsequent RCT, we planned to collect EMG data only for a limited subgroup of participants (for more detail, see the study protocol of the RCT [47]).

Results of the manipulation check suggest that the negative mood induction successfully induced depressed mood with a moderate-to-large effect size (Hedges *g*=–0.69), and reduced positive mood with a large effect size (Hedges *g*=–0.95). These results confirm the validity of the mood induction procedure [56,57] and its usefulness in the study of depression interventions. With regards to the clinical potential, rejecting the depressogenic statements in the first training phase and approving the antidepressive statements in the second training phase did not affect the depressed mood. Positive mood, however, did increase with a moderate-to-large effect (Hedges *g*=–0.63) in the first training phase and a small effect (Hedges *g=*–0.25) in the second training phase. The null findings for depressed mood may be due to floor effects for our healthy feasibility sample as the mean rating of depressed mood was very low throughout data collection, even after the negative mood induction and the presentation of the depressogenic statements. This is in line with results from the study of O’Toole and Michalak [46], who investigated the effects of augmenting CR with antidepressive body posture and movement. As in our study, healthy individuals were included and no effects on negative emotions were found. Findings from the study by Stiles-Shields et al, however, evaluating the effects of a structured cognitive restructuring exercise, were the opposite, with significant effects on symptoms of depression [24]. With regard to previous studies investigating smartphone-based CR augmented by antidepressive kinesthesia, the findings were similar. One of the studies examining AAMT focusing on CR found significant effects on symptoms of depression [38] (the other reported only descriptive data [39]). These differences may be explained by the fact that Stiles-Shields et al [24] as well as Lukas et al [38] examined individuals with symptoms of depression in their studies and not healthy individuals as in our study. Furthermore, these studies assessed symptoms of depression over the past week, whereas this study assessed the intervention’s immediate effect on mood. Moreover, both studies assessed various symptoms of depression, while the current study focused exclusively on depressed mood.

The findings for positive mood, however, align with our hypothesis, suggesting that counteracting depressogenic statements in particular with antidepressive statements and kinesthesia exercises has positive effects on mood in healthy individuals. As such, they are in line with studies showing positive effects of CR on mood, such as those included in a meta-analysis by Ciharova et al [11]. In summary, the results of this and previous studies are promising with regard to the clinical potential of a structured cognitive restructuring exercise as well as the potential of augmenting cognitive restructuring with antidepressive kinesthesia. These findings are particularly important because structured cognitive restructuring exercises, which also lead to emotional insight through augmentation with antidepressive kinesthesia, could easily be used in mobile interventions without direct human interaction. The types of kinesthesia that are particularly beneficial and the extent to which they augment effects of structured cognitive restructuring on depressed and positive mood as posited by the ICS theory of depression [25] and studies demonstrating effects of manipulating facial expressions on depressed mood [29,30,34,36], will be subject of our subsequent RCT.

Beyond investigating the feasibility and clinical potential of the training, this feasibility study enabled us the opportunity to ensure that the technical aspects of the study (ie, the kinesthetic and physiological data acquisition) ran smoothly. In this respect, we have drawn several implications from the feasibility results. For instance, to improve the kinesthetic data acquisition, we decided to use a second high-resolution RGB camera in the upcoming RCT to record participants’ upper bodies and heads viewed from the side. Moreover, after evaluating our data and consulting with experts in the field, contrary to our initial preregistrations (RCT [76]; feasibility study [48]), we decided to drop the electrodermal activity and pupillometry measurements, as well as the EMG measures of the trapezius due to poor data quality and because an improvement of data quality would only have been possible at the cost of disrupting therapeutic processes. Overall, all further issues jeopardizing the quality of kinesthetic and psychophysiological measures could be fixed before the RCTs start, thereby optimizing the development of machine-learning models for automated depression assessment.

### Limitations and Conclusion

Major limitations of this feasibility study include the fixed sequence of first invalidating depressogenic cognitions and thereafter validating antidepressive cognitions. This harbors the risk of position effects on depressed and positive moods. Therefore, we plan to randomize the order of the 2 training phases in the RCT. In addition, the intensive contact with study therapists during the training may have prompted socially desirable behavior in participants, particularly regarding compliance with the training and mood ratings submitted during the training. Future studies should include measures of social desirability and ways to control its effects. On the other hand, this contact also represents a strength of the study, as the therapeutic relationship is used to increase the efficacy of the intervention. Despite these limitations and the small sample size, this feasibility study provided valuable insights that led to significant improvements in the study protocol of our upcoming RCT. As such, it serves as a critical keystone in the further development and investigation of innovative interventions for depression.

## Appendix

Multimedia Appendix 1 American Psychological Association journal article reporting standards.

Multimedia Appendix 2 Self-developed items to assess usability and acceptability.

## Abbreviations

AAMT: approach-avoidance modification training
AFE: antidepressive facial expression
CBT: cognitive behavioral therapy
CR: cognitive reappraisal
EMG: electromyography
EmpkinS: Empatho-Kinaesthetic Sensor Technology
FAU: Friedrich-Alexander-Universität Erlangen-Nürnberg
GRID-HAMD: GRID Hamilton Depression Rating Scale
ICS: interacting cognitive subsystems
PHQ: Patient Health Questionnaire
RCT: randomized controlled trial
SCID-5-CV: Structured Clinical Interview for DSM-5 Disorders–Clinician version
UEQ-S: Short Version of the User Experience Questionnaire

## Data Availability

The data sets analyzed during this study and the analysis code are available in the Open Science Framework [48].
