# Supplementary material for: The EmpkinS-EKSpression Reappraisal Training Augmented With Kinesthesia in Depression: One-Armed Feasibility Study
Source: JMIR Form Res. 2025 Apr 14;9:e65357. doi: 10.2196/65357 (PMC12038297; doi:10.2196/65357)
Supplement: Multimedia Appendix 1 [file formative_v9i1e65357_app1.pdf]

APA Style **JARS**

Journal Article Reporting Standards

JARS–**Quant** | Table 1
Information Recommended for Inclusion in Manuscripts That Report New Data Collections Regardless of Research Design

Title and Title Page

Title

- Identify main variables and theoretical issues under investigation and the relationships between them.
- Identify the populations studied.

The EmpkinS-EKSpression Reappraisal Training Augmented with Kinesthesia in Depression: One-Armed Feasibility Study

Author Note

- Provide acknowledgment and explanation of any special circumstances, including
  - registration information if the study has been registered
  - use of data also appearing in previous publications
  - prior reporting of the fundamental data in dissertations or conference papers
  - sources of funding or other support
  - relationships or affiliations that may be perceived as conflicts of interest
  - previous (or current) affiliation of authors if different from location where the study was conducted
  - contact information for the corresponding author
  - additional information of importance to the reader that may not be appropriately included in other sections of the paper

Not in line with journal requirements

Abstract

Objectives

- State the problem under investigation, including main hypotheses.

This study aimed to examine the feasibility and clinical potential of a smartphone-based cognitive restructuring task that required users to deliberately perform antidepressive kinesthesia in conjunction with the rejection of depressogenic statements and the affirmation of antidepressive statements. This feasibility study was conducted as a precursor to a large-scale randomized controlled trial.

Participants

- Describe subjects (nonhuman animal research) or participants (human research), specifying their pertinent characteristics for the study; in animal research, include genus and species. Participants are described in greater detail in the body of the paper.

10 healthy participants

Study Method

- Describe the study method, including
  - research design (e.g., experiment, observational study)
  - sample size
  - materials used (e.g., instruments, apparatus)
  - outcome measures
  - data-gathering procedures, including a brief description of the source of any secondary data. If the study is a secondary data analysis, so indicate.

In total, 10 healthy participants engaged in a single 90-120-minute session of smartphone-based CR training. During the training, they completed 2 phases in which they were required to reject 20 depressogenic and affirm 20 anti-depressive statements, respectively. Diagnostic assessments were conducted 1 week (T1) before and directly prior (T2) to the training, and again directly post-training (T3) and at 2-week follow-up post-training (T4). Feasibility outcomes assessed at T3 included intervention safety recorded by study therapists, compliance, technical feasibility, usability assessed using the User Experience Questionnaire – short form (UEQ-S), and acceptability assessed using the UEQ-S and self-developed items. Preliminary clinical potential was evaluated via single-item ratings of current depressed and positive mood assessed continuously during the training. Feasibility outcomes were analyzed descriptively, and clinical potential was examined using paired-samples *t*-tests of pre and post mood ratings of each training phase.

Findings

- Report findings, including effect sizes and confidence intervals or statistical significance levels.

Overall, the results indicated that the training was safe, feasible, and usable (UEQ-S pragmatic quality scale: mean 1.45, SD 0.71). However, acceptance was limited (UEQ-S hedonic quality scale: mean 1.05, SD 0.79). While 80% (8/10) of the participants were generally satisfied with the training, 80% (8/10) would recommend it to a friend, 90% (9/10) found it interesting, and 80% (8/10) rated it as “leading edge,” 40% (4/10) to 70% (7/10) did not consider it particularly helpful and 50% (5/10) found it repetitive. Preliminary results regarding clinical potential were promising, with significant increases in positive mood (rejection: Hedges g=0.63; affirmation: Hedges g=0.25), whereas changes in depressed mood were not significant.

Conclusions

- State conclusions, beyond just results, and report the implications or applications.

This study evaluated the feasibility and acceptability of a smartphone-based cognitive reappraisal training augmented with (in-)validating kinesthesia. This provided invaluable insights for further optimizing the intervention for the subsequent RCT, but also potential similar interventions. If future studies confirm their clinical potential, such interventions offer a promising approach to enhance CBT for depression.

Introduction

Problem

- State the importance of the problem, including theoretical or practical implications.

Depression is one of the most prevalent mental disorders [1] and severely impacts quality of life and functioning of affected individuals [2]. Depression is also associated with an increased risk of cardiovascular disease [3] and mortality [4]. Although Cognitive Behavioral Therapy (CBT) has been shown to be effective in depression treatment [5], dropout rates of up to 32 % [6], low remission rates of 42 % [7], and low rates of evidence-based treatment administration [8] highlight the need to further optimize CBT for depression.
CBT for depression is based on Beck’s cognitive model of depression [9], which assumes that dysfunctional beliefs (e.g., “I am worthless”) are a key factor in the etiology and maintenance of the disease. Therefore, one primary goal of CBT is the reappraisal of such dysfunctional beliefs. This is typically pursued with cognitive restructuring techniques, with which therapists guide patients to question the validity of their dysfunctional beliefs and to articulate more adaptive ones [9]. Theory and empirical evidence suggest that invalidating dysfunctional beliefs while strengthening functional beliefs has positive effects on depressed mood and other symptoms of depression [10,11]. To

reduce the large treatment gap in depression, researchers have developed accessible and cost-effective CBT-based app interventions that include cognitive restructuring exercises, amongst others. Such interventions have proven to be effective in reducing symptoms of depression [12]. However, a meta-analysis evaluating single compontents of such interventions with individual participant data found no clear evidence for the efficacy of cognitive restructuring when used within digital interventions [13], highlighting the need to optimize digital cognitive restructuring interventions.

Review of Relevant Scholarship

- Provide a succinct review of relevant scholarship, including
  - relation to previous work
  - differences between the current report and earlier reports if some aspects of this study have been reported on previously

Most of the mobile cognitive restructuring exercises in the context of depression take a similar approach as therapists in face-to-face therapy: They offer psychoeducational content about the relationship between dysfunctional beliefs and depression and provide Socratic questions to help individuals invalidate dysfunctional beliefs and develop more adaptive ones (e.g., [14–19]). However, without the support of a therapist, this approach could be very challenging for patients, especially in the context of cognitive and motivational impairments in depression [20,21]. This is also supported by pilot results indicating lower adherence rates with a cognitive restructuring intervention than with an intervention focusing on behavioral activation [18]. Thus, in order to increase adherence, a structured cognitive restructuring exercise with pre-selected content might be of greater benefit in the context of app interventions without direct human interaction. To our knowledge, only two studies evaluate such an intervention. McCloud and colleagues [22] propose a cognitive restructuring exercise based on the ABC-technique [23] providing options of alternative interpretations for users to choose from. Stiles-Shields and colleagues [24] propose a step-by-step procedure for identifying and changing maladaptive thoughts using Socratic questions, providing examples of possible thoughts and alternatives. However, the efficacy of McCloud and colleagues' [22] cognitive restructuring exercise cannot be determined as it was part of a multi-component intervention. Stiles-Shields and colleagues [24] conducted a pilot trial. Although they did find effects on symptoms of depression, the informative value is limited due to the small sample size. The efficacy of such structured cognitive restructuring exercises therefore still requires empirical support. […]

[F]ew studies so far have investigated the effects of augmenting cognitive interventions and, more specifically, cognitive reappraisal interventions with (in-)validating kinesthesia. Adaptations of the approach-avoidance-modification-training (AAMT), which originally required participants to push/pull joysticks to move disorder-relevant images away from/towards themselves [37], use disorder-relevant dysfunctional beliefs as stimuli [38–45]. Two of the studies following such an approach addressed depression (i.e., with stimuli such as "I am a failure"), with promising effects on symptoms of depression [38,39]. Moreover, pilot findings from the stress context suggest that using the deliberate display of positive and negative emotions through facial expression, body posture, and a corresponding statement as responses within AAMT might enhance its efficacy [43–45]. Another, albeit not smartphone-based study by O'Toole and Michalak [46] followed an approach more closely aligned with cognitive restructuring in CBT. They evaluated a cognitive restructuring exercise delivered face-to-face combined with emotion-focused body postures and movements and found that it produced greater decreases in agreement with dysfunctional beliefs than isolated cognitive restructuring in healthy individuals.

Hypothesis, Aims, and Objectives

State specific hypotheses, aims, and objectives, including

- theories or other means used to derive hypotheses
- primary and secondary hypotheses
- other planned analyses

- State how hypotheses and research design relate to one another

Although these approaches are promising, evidence remains preliminary, particularly in the context of depression treatment and no study so far has evaluated the efficacy of a smartphone-based structured cognitive restructuring exercise enhanced with (in-)validating kinesthesia. To advance research in this regard, we developed a structured smartphone-based and sensor-supported cognitive reappraisal intervention, in which depressogenic verbalizations are paired with invalidating facial expressions, and anti-depressive verbalizations are paired with validating facial expressions. Our ultimate goal is to train machine-learning models in the automated assessment of depression, which could then be integrated into a fully-automated, smartphone-based biofeedback training. The primary goal of the current study was to evaluate the feasibility and safety of this intervention in a limited healthy sample by testing its technical setup and procedures before implementation of a large-scale randomized controlled trial (RCT) involving participants with and without depression (for the study protocol of the RCT, see [47]). Second, we aimed to gain preliminary insights into the clinical potential of the intervention, expecting an overall decrease in depressed mood and an increase in positive mood throughout the training and beyond.

Specific hypotheses are not applicable due to the feasibility character of the study

Method

Inclusion and Exclusion

- Report inclusion and exclusion criteria, including any restrictions based on demographic characteristics

Inclusion criteria were (1) minimum age 18 years and (2) sufficient German language skills (at least level B2 according to the Common European Framework of Reference for Languages). Exclusion criteria were (1) a Patient Health Questionnaire (PHQ)-8 score of ≥ 4, (2) sufficient criteria for any mental disorder (ICD-10 F1-F9), (3) acute suicidality, (4) any impairment of facial expression, and (5) dyschromatopsia.

Participant Characteristics

- Report major demographic characteristics (e.g., age, sex, ethnicity, socioeconomic status) and important topic-specific characteristics (e.g., achievement level in studies of educational interventions).
- In the case of animal research, report the genus, species, and strain number or other specific identification, such as the name and location of the supplier and the stock designation. Give the number of animals and the animals' sex, age, weight, physiological condition, genetic modification status, genotype, health–immune status, drug or test naïveté, and previous procedures to which the animal may have been subjected.

See Table 1 in the manuscript

Sampling Procedures

- Describe procedures for selecting participants, including
  - sampling method if a systematic sampling plan was implemented
  - percentage of sample approached that actually participated
  - whether self-selection into the study occurred (either by individuals or by units, such as schools or clinics)

study screening questionnaire assessing inclusion and exclusion criteria, as well as symptoms of depression with the Patient Health Questionnaire (PHQ)-8 [52] and contact information. Eligible participants were contacted via email or telephone and invited to a diagnostic session (T1). All on-site study sessions were held at the EmpkinS Lab of the Friedrich-Alexander-Universität Erlangen-Nürnberg. The laboratory rooms were located in a scientific university building and set up specifically for this study. At the beginning of the diagnostic session participants provided written

- Describe settings and locations where data were collected as well as dates of data collection.
- Describe agreements and payments made to participants.
- Describe institutional review board agreements, ethical standards met, and safety monitoring.

informed consent. We then conducted the Structured Clinical Interview for DSM-5 Disorders - Clinician version (SCID-5-CV; German version: [53]) to assess the diagnostic status of participants. One week after the diagnostic session, the training session was held at the Lab.. The entire session took about 3 hours.
Two weeks after the training session, participants were invited to a follow-up assessment (T4) via telephone and online questionnaire.
Participants received compensation up to 40€ (10€ for the diagnostic session, 20€ for the training session, 5€ for the follow-up assessment, and a 5€ bonus for completing the whole study). Psychology students of FAU could alternatively receive course credits.
The study followed the Declaration of Helsinki's ethical guidelines and obtained ethical approval by FAU's Ethics committee (20-443-B, 20-443_1-B, 20-443_2-B). All participants provided written informed consent
See also Figure 2 in the manuscript for the participant flow throughout the study

Sample Size, Power, and Precision

- Describe the sample size, power, and precision, including
  - intended sample size
  - achieved sample size, if different from the intended sample size
  - determination of sample size, including
    - power analysis, or methods used to determine precision of parameter estimates
    - explanation of any interim analyses and stopping rules employed

We aimed to include *N* = 10 participants, following heuristics from the literature concerning optimal sample sizes for feasibility studies [50,51] and internal considerations of practicality and time constraints.
See also Figure 2 in the manuscript for the participant flow throughout the study

Measures and Covariates

- Define all primary and secondary measures and covariates, including measures collected but not included in the report.

To determine the feasibility of the intervention, we assessed safety, technical feasibility, compliance, usability, and acceptability.
The primary clinical outcome was current depressed mood and the secondary outcome was current positive mood.
Demographic and health-related variables included age, date of birth, German language skills, nationality, gender, height, weight, academic degree, professional occupation, relationship status, scholarly fields of study (if any), current/prior diagnosis of a mental illness, psychotherapy, medication, color-blindness, and Botox-treatment. In addition, the PHQ-8 [52] was used to assess current depressive symptom severity during diagnostic screening. The PHQ-8 consists of eight items assessing the frequency of eight of the nine DSM-criteria of depression during the prior two weeks on a four-point Likert-scale (0 = *not at all* to 3 = *nearly every day*).
As one aim of our feasibility study was to test the technical setup of the study and protocol procedures, we included all measures to be assessed in the RCT. However, the additional measures listed below will not be analyzed in this feasibility evaluation. Clinical status of participants was assessed with the SCID-5-CV during diagnostic screening. Symptoms of depression were assessed with the GRID-HAMD [54] during the training session and at follow-up. In addition, self-reported symptoms of depression were assessed with the Centre for Epidemiological Studies Depression Scale (German version [63]). Current suicidality was assessed with a single self-developed item. Dysfunctional attitudes were assessed with the German short version of the Dysfunctional Attitude Scale (DAS), form A [64], and automatic thoughts with the German version of the Automatic Thoughts Questionnaire-Revised (ATQ-R [60]). Finally, emotional state was assessed with the Self-Report Instrument for the Assessment of Emotion-Specific Regulation Skills parts A and B (SEK-ES

[65]). Two video cameras were used to assess kinesthesia (i.e., facial expression and body posture): an Azure Kinect depth camera (Microsoft, Redmond, WA, USA) and a high-resolution RGB camera (Sony SRG-300H), positioned 1.2 meters in front of participants. In addition, bipolar EMG of various facial muscles (i.e., M. corrugator supercilii, M. zygomaticus major, M. masseter, M. orbicularis oculi) and of the shoulder muscle (M. trapezius) were assessed with the BioPac MP160 system (Biopac Inc. Goleta, CA, USA). Further psychophysiological measures assessed with the BioPac MP160 system included electrocardiogram, respiration (using a respiration belt), and electrodermal activity (EDA). In addition, heart rate and respiration were assessed with radar-based technology [66]. The preparatory phase served as a baseline period for physiological data acquisition. Further, we assessed pupillometry data. Moreover, the smartphone app recorded videos of each trial and participants' digital activity on the app using the smartphone's front camera.

Data Collection

- Describe methods used to collect data.

We then conducted the Structured Clinical Interview for DSM-5 Disorders - Clinician version (SCID-5-CV; German version: [53]) to assess the diagnostic status of participants. After the interview, participants answered a set of questionnaires via Unipark.com (T1).
Two weeks after the training session, participants were invited to a follow-up assessment (T4). The GRID-HAMD was conducted via telephone and participants were asked to fill out an online questionnaire via Unipark.com. They received the link to the questionnaires via email one day before the telephone interview.

Quality of Measurements

- Describe methods used to enhance the quality of measurements, including
  - training and reliability of data collectors
  - use of multiple observations

The assessments and study sessions were conducted by trained clinical psychologists

Instrumentation

- Provide information on validated or ad hoc instruments created for individual studies, for individual studies (e.g., psychometric and biometric properties).

Safety of the intervention: To determine the safety of the intervention, study therapists recorded whether participants experienced significant mood deterioration during the training. The intervention was deemed safe if no more than 10% of the training sessions were prematurely terminated due to significant mood deterioration.
Compliance. The compliance rate was defined as the number of participants completing the intervention per protocol. The intervention was considered feasible if the compliance rate was at least 50%.
Technical feasibility. Technical difficulties with the EmpkinS-EKSpression app during the training were to be reported by study therapists. We considered the intervention feasible if at least 80% of the sessions were completed without major technical problems.
Usability. The usability of the training app was assessed with the pragmatic quality scale of the User Experience Questionnaire – short form (UEQ-S [62]) and with one self-constructed item. In the UEQ-S, eight pairs of opposite adjectives related to user experience are rated on a seven-point Likert scale from -3 (*fully agree with negative term*) to +3 (*fully agree with positive term*). The pragmatic quality scale consists of four of the eight items, with the other four items belonging to the hedonic quality scale. The self-constructed item asked participants how intelligible the app-instructions were on a four-point Likert scale from 1 (*not at all*) to 4 (*very*). We considered the intervention usable if >80% of participants rated the app as positive (score ≥ 1), i.e., supportive (rather than obstructive), easy (rather than complicated), efficient (rather than inefficient), clear

(rather than confusing), and very intelligible.
Acceptability. Acceptability of the intervention was assessed with the 'hedonic quality' scale of the UEQ-S. The intervention was considered acceptable if >80% of participants rated the training as positive (score ≥ 1), i.e., interesting (vs. not interesting), exciting (vs. boring), inventive (vs. conventional), and leading edge (vs. usual). Twelve additional self-generated items were used to assess acceptability (see supplementary materials for the items). Except for two items, they were all rated on a four-point Likert scale from 1 (*not at all*) to 4 (*very*). One item asking whether participants would recommend the training to a friend was answered with *yes*/*no*. Another item asking whether the training takes a reasonable amount of time was rated on a five-point Likert scale from 1 (*far too high*) to 5 (*far too low*). The intervention was considered acceptable if >80% of participants indicated they would recommend the training to a friend, were rather content with the training, would continue the training given the opportunity, considered the training as rather helpful, and fun (score ≥ 3), as well as requiring a reasonably acceptable amount of time (score 2-4), and rate the training as only little or not at all strenuous and difficult to focus on (score ≤ 2). Moreover, open-ended questions were asked by study therapists during the training and at the T3 assessment to gain additional, qualitative feedback for improvement. Acceptability of the overall study was assessed with one item asking participants to rate how burdensome study participation had been on a four-point Likert scale from 1 (*not at all*) to 4 (*very*). The study was considered acceptable if >80% of participants rated participation as only a little or not at all burdensome.
Current depressed mood was assessed using an eleven-point Likert-scale from 0 (*no depressed mood at all*) to 10 (*very strong depressed mood*) during both diagnostic and training sessions. During training, participants submitted 108 ratings in total: 24 during the preparatory phase, one after the negative mood induction, one immediately before the first training item, one after each initial statement, and one after the completion of each training trial.
Current positive mood was also assessed using an eleven-point Likert-scale from 0 (*no positive mood at all*) to 10 (*very strong positive mood*). Similar to the number of ratings of depressed mood, participants submitted a total of 108 ratings of positive mood.

Masking

- Report whether participants, those administering the experimental manipulations, and those assessing the outcomes were aware of condition assignments.
- If masking took place, provide a statement regarding how it was accomplished and whether and how the success of masking was evaluated.

Not applicable

Psychometrics

- Estimate and report values of reliability coefficients for the scores analyzed (i.e., the researcher's sample), if possible. Provide estimates of convergent and discriminant validity where relevant.
- Report estimates related to the reliability of measures, including
  - interrater reliability for subjectively scored measures and ratings
  - test–retest coefficients in longitudinal studies in which the retest interval corresponds to the measurement schedule used in the study
  - internal consistency coefficients for composite scales in which these indices are appropriate for understanding the nature of the instruments being used in the study

Not applicable

- Report the basic demographic characteristics of other samples if reporting reliability or validity coefficients from those samples, such as those described in test manuals or in norming information for the instrument.

Conditions and Design

- State whether conditions were manipulated or naturally observed. Report the type of design as per the JARS–Quant tables:
  - experimental manipulation with participants randomized
    - › Table 2 and Module A
  - experimental manipulation without randomization
    - › Table 2 and Module B
  - clinical trial with randomization
    - › Table 2 and Modules A and C
  - clinical trial without randomization
    - › Table 2 and Modules B and C
  - nonexperimental design (i.e., no experimental manipulation): observational design, epidemiological design, natural history, and so forth (single-group designs or multiple- group comparisons)
    - › Table 3
  - longitudinal design
    - › Table 4
  - *N*-of-1 studies
    - › Table 5
  - replications
    - › Table 6

Report the common name given to designs not currently covered in JARS–Quant

See JARS-Quant Table 2

Data Diagnostics

- Describe planned data diagnostics, including
  - criteria for post-data-collection exclusion of participants, if any
  - criteria for deciding when to infer missing data and methods used for imputation of missing data
  - definition and processing of statistical outliers
  - analyses of data distributions
  - data transformations to be used, if any

Not applicable

Analytic Strategy

- Describe the analytic strategy for inferential statistics and protection against experiment- wise error for
  - primary hypotheses
  - secondary hypotheses
  - exploratory hypotheses

The safety and feasibility of the intervention and collection of kinaesthetic and physiological data were determined based on descriptive analyses. Results were compared to predefined threshold values (see above). As a manipulation check, we analyzed whether the negative mood induction successfully elicited an increase in depressed mood and/or a decrease in positive mood. To this end, a paired-samples *t*-test was computed to compare depressed/positive mood before and after the negative mood induction. To analyze the clinical potential of the intervention, we also computed

paired-samples *t*-tests to compare the mean rating of depressed (dependent variable 1) and positive mood (dependent variable 2) *after presentation of the statements*, with the mean depressed and positive mood rating *after completion of the trials*. We analyzed the clinical potential separately for the two training phases. For effect sizes, we computed Hedge's *g* as proposed by Lakens [67] for small samples, with g = 0.2/0.5/0.8 as small/moderate/large effect [68]. Moreover, we implemented two linear mixed-effects models with time-point as fixed effect on level 1 and random intercepts and random slopes to analyze the course of depressed and positive mood ratings over the course of the training. The dependent variables were depressed mood and positive mood, respectively. However, as our data showed too little variance, particularly during the second training phase, the models appeared to not converge. Therefore, we merely analyzed the course of depressed and positive mood on a descriptive level.
The level of significance for all analyses was α = .05. The analyses were conducted with R Studio, version 4.3.2 [69].

Results

Participant Flow

- Report the flow of participants, including
  - total number of participants in each group at each stage of the study
  - flow of participants through each stage of the study (include figure depicting flow, when possible; see the JARS–Quant Participant Flowchart)

See Figure 2 in the manuscript for the participant flow throughout the study

Recruitment

- Provide dates defining the periods of recruitment and repeated measures or follow-up.

Participants were recruited between August 2022 and January 2023.

Statistics and Data Analysis

- Provide information detailing the statistical and data-analytic methods used, including
  - missing data
    - frequency or percentages of missing data
    - empirical evidence and/or theoretical arguments for the causes of data that are missing—for example, missing completely at random (MCAR), missing at random (MAR), or missing not at random (MNAR)
    - methods actually used for addressing missing data, if any
  - descriptions of each primary and secondary outcome, including the total sample and each subgroup, that includes the number of cases, cell means, standard deviations, and other measures that characterize the data used
  - inferential statistics, including
    - results of all inferential tests conducted, including exact *p* values if null hypothesis significance testing (NHST) methods were used, and reporting the minimally sufficient set of statistics (e.g., *df*s, mean square [*MS*] effect, *MS* error) needed to construct the tests
    - effect-size estimates and confidence intervals on estimates that correspond to each inferential test conducted, when possible
    - clear differentiation between primary hypotheses and their tests–estimates,

Regarding safety and compliance, none of the participants terminated the training prematurely. Technical difficulties with the EmpkinS-EKSpression app occurred in two training sessions: One of them had to be terminated immediately after the start due to an error in the app, leading to the drop-out of that participant (see Fig. 1). The other session was terminated after the first training phase also due to an error in the app. Here, the data recorded by the smartphone app (including mood ratings delivered throughout the training) were missing. Therefore, this participant was not included in the analysis of clinical potential. However, the data were included in the feasibility analyses.
With regards to usability as assessed with the pragmatic quality scale of the UEQ-S, favorable results emerged. Except for the instrument's "complicated – easy" item, all items were rated positively (see Table 2). The total score of the pragmatic quality scale was *M* = 1.45 (*SD* = 0.71). 70% (7/10) of participants assessed the overall usability positively. Ratings for our self-developed item on the intelligibility of the app instructions ranged from 3 to 4 with a median of 4 (*M* = 3.9, *SD* = 0.32). 90% (9/10) of participants rated the instructions as very intelligible.
For acceptability, as assessed with the hedonic quality scale of the UEQ-S, results were more mixed (see Table 2). While the total score was *M* = 1.05 (*SD* = 0.79), only 50% (5/10) of participants reached a positive hedonic quality scale total score. Regarding our self-developed items, 80% (8/10) of participants indicated they would recommend the training to a friend. Further descriptive statistics are displayed in Table 3. In the open evaluation of the training, four participants revealed that they found the training repetitive; two found it exhausting. Five participants found the emotion display, three pride

secondary hypotheses and their tests–estimates, and exploratory hypotheses and their test–estimates

- complex data analyses—for example, structural equation modeling analyses (see also Table 7), hierarchical linear models, factor analysis, multivariate analyses, and so forth, including
  - details of the models estimated
  - associated variance–covariance (or correlation) matrix or matrices
  - identification of the statistical software used to run the analyses (e.g., SAS PROC GLM or the particular R package)
- estimation problems (e.g., failure to converge, bad solution spaces), regression diagnostics, or analytic anomalies that were detected and solutions to those problems.
- other data analyses performed, including adjusted analyses, if performed, indicating those that were planned and those that were not planned (though not necessarily in the level of detail of primary analyses).

• Report any problems with statistical assumptions and/or data distributions that could affect the validity of findings.

in particular, initially unfamiliar and difficult to engage in. Moreover, five participants were critical that the standardized statements were not better tailored for individuals, and three were dissatisfied with the study setting (i.e., uncomfortable seating position, feeling observed by the study therapist, and awkward electrodes). In contrast, some positive feedback included that the training was fun and interesting (n = 4), that it provided new ideas for dealing with negative thoughts (n = 4), and that there were several statements to choose from (n = 1). Regarding acceptability of the study, participation burden ratings ranged from 1 to 2, with a median of 1 (M = 1.1, SD = 0.2), hence, 100% (10/10) of participants rated the participation as only a little or not at all burdensome.

See also Tables 2-3 in the manuscript

With regards to negative mood induction, the mean rating of depressed mood was M = 0.5 (SD = 1.41) before and M = 1.44 (SD = 0.73) after mood induction. The mean rating of positive mood was M = 5.67 (SD = 1.66) before and M = 4.56 (SD = 1.88) after negative mood induction. Both paired-samples t-test reached significance (t(7) = -3.06, p = .02, g = -0.69, 95% CI [-2.83, -0.28] and t(8) = 3.16, p = .01, g = -0.95, 95% CI [-1.84, -0.55], respectively).

In the first training phase, the mean rating of depressed mood was M = 2.63 (SD = 2.51) before and M = 2.41 (SD = 2.36) after, and the mean rating of positive mood was M = 4.14 (SD = 2.42) before and M = 5.59 (SD = 1.65) after the explicit rejection of depressogenic statements, respectively. The difference was not significant for depressed mood (t(178) = 1.16, p = .25, g = 0.09, 95% CI [-0.07, 0.23]), but did reach significance for positive mood (t(179) = -8.53, p < .001, g = -0.63, 95% CI [-0.72, -0.55]). In the second training phase, the mean rating of depressed mood was M = 2.75 (SD = 2.47) before and M = 2.74 (SD = 2.48) after, and the mean rating of positive mood was M = 5.62 (SD = 1.71) before and M = 5.81 (SD = 1.58) after the explicit substantiation of anti-depressive statements, respectively. The difference was not significant for depressed mood (t(178) = -0.16, p = .87, g = -0.01, 95% CI [-0.16, 0.12]), but did, again, reach significance for positive mood (t(177) = -3.38, p < .001, g = -0.25, 95% CI [-0.38, -0.11]). Descriptively, there was a visible mood change after the negative mood induction (i.e., an increase in depressed, and a decrease in positive mood) and an increase in positive mood after each trial, particularly during the first training phase (see Figure 3). Moreover, there was a slight increase in depressed mood over the course of the first training phase, and in positive mood over the course of the second training phase.

Discussion

Support of Original Hypotheses

• Provide a statement of support or nonsupport for all hypotheses, whether primary or secondary, including
- distinction by primary and secondary hypotheses
- discussion of the implications of exploratory analyses in terms of both substantive findings and error rates that may be uncontrolled

With regard to feasibility, the results were quite positive, and the predefined benchmarks were met for safety, compliance, and technical feasibility. Concerning the usability of the training app as assessed with the pragmatic quality scale of the UEQ-S and a self-developed item on intelligibility of the app instructions, the predefined benchmark was narrowly missed.
Results on the acceptability of the training, as assessed with the hedonic quality of the UEQ-S, as well as several self-constructed items, did not meet the predefined benchmarks.
Results of the manipulation check suggest that the negative mood induction successfully induced depressed mood with a moderate-to-large effect size (g = -0.69), and reduced positive mood with a large effect size (g = -0.95). These results confirm the validity of the mood induction procedure [56,57] and its usefulness in the study of depression interventions. With regards to the clinical potential, rejecting the depressogenic statement in the first training phase and approving the anti-

depressive statement in the second training phase did not affect depressed mood. Positive mood, however, did increase with a moderate-to-large effect (*g* = -0.63) in the first training phase and a small effect (*g* = -0.25) in the second training phase.

Similarity of Results

- Discuss similarities and differences between reported results and work of others.

This result is worse than the findings of Stiles-Shields and colleagues who evaluated a structured cognitive restructuring exercise where usability was rated to be high [24]. An important, possibliy explanatory difference in this study is a higher degree of individualization: participants could choose between self-generated and pre-selected content in each step of the cognitive restructuring task. It is possible that including more options for individualization of content might improve the usability of the EmpkinS-EKSpression training. Similarly, one of the studies investigating an AAMT intervention in the context of depression reported high usability ratings [39]. The training content here was highly standardized, so a lack of individualization might not be the only explanatory factor for missing the usability benchmark in this study.
One difference was the amount of time spent on the cognitive restructuring task (which was not reported by Stiles-Shields and colleagues [24]). While individuals in our study trained for 90-120 minutes, individuals in the study by Lukas and Berking completed an average of around 40 minutes of training [39].
This finding is in line with previous work investigating the acceptability of a digital cognitive bias modification training that also required participants to complete many trials, which suggest that participants' willingness to engage in repetitive digital treatments is limited [70].
This is in line with results from the study of O'Toole and Michalak [46], who investigated the effects of augmenting cognitive reappraisal with anti-depressive body posture and movement. As in our study, healthy individuals were included and no effects on negative emotions were found. Findings from the study by Stiles-Shields and colleagues, however, evaluating the effects of a structured cognitive restructuring exercise, were opposite, with significant effects on symptoms of depression [24]. With regard to previous studies investigating smartphone-based cognitive reappraisal augmented by anti-depressive kinesthesia, findings were similar. One of the studies examining AAMT focusing on cognitive reappraisal found significant effects on symptoms of depression [38] (the other reported only descriptive data [39]). These differences may be explained by the fact that Stiles-Shields and colleagues [24] as well as Lukas and collagues [38] examined individuals with symptoms of depression in their studies and not healthy individuals as in our study. Furthermore, these studies assessed symptoms of depression over the past week, whereas the current study assessed the intervention's immediate effect on mood. Moreover, both studies assessed various symptoms of depression, while the current study focused exclusively on depressed mood.

Interpretation

- Provide an interpretation of the results, taking into account
  - sources of potential bias and threats to internal and statistical validity
  - imprecision of measurement protocols
  - overall number of tests or overlap among tests
  - adequacy of sample sizes and sampling validity

Nevertheless, the explicit display of anti-depressive kinesthesia might be more challenging than mere cognitive restructuring exercises
Our feasibility study was likely to fall short of meeting this requirement, given that we only included healthy individuals with perhaps only limited need for a mood enhancement intervention. Thus, the content of the training was probably not sufficiently relevant for participants, which in turn could have compromised its credibility.
The fact that our feasibility sample consisted of healthy individuals might also explain the finding that participants did not consider the training to be particularly helpful. If they did not relate to the standardized depressogenic statements in the first place (which reflected in participants' open

feedback), then there were no negative underlying cognitions to be modified.
The null findings for depressed mood may be due to floor effects for our healthy feasibility sample as the mean rating of depressed mood was very low throughout data collection, even after the negative mood induction and the presentation of the depressogenic statements.
The findings for positive mood, however, align with our hypothesis, suggesting that particularly counteracting depressogenic statements with anti-depressive statements and kinesthesia exercises positive effects on mood in healthy individuals.

Generalizability

- Discuss generalizability (external validity) of the findings, taking into account
  - target population (sampling validity)
  - other contextual issues (setting, measurement, time; ecological validity)

Our feasibility study was likely to fall short of meeting this requirement, given that we only included healthy individuals with perhaps only limited need for a mood enhancement intervention. Thus, the content of the training was probably not sufficiently relevant for participants, which in turn could have compromised its credibility. However, it is common practice for feasibility studies to initially scrutinize general intervention effects in healthy individuals before exposing more vulnerable clinical participants to those effects [72].
Major limitations of this feasibility study include the fixed sequence of first invalidating depressogenic cognitions and thereafter validating anti-depressive cognitions. This harbors the risk of position effects on depressed and positive mood.
In addition, the intensive contact with study therapists during the training may have prompted socially desirable behavior in participants, particularly regarding compliance with the training and mood ratings submitted during the training.

Implications

- Discuss implications for future research, program, or policy.

It is possible that including more options for individualization of content might improve the usability of the EmpkinS-EKSpression training.
Therefore, a shorter treatment duration might be beneficial to increase usability.
Therefore, this finding should be considered in future studies using similar paradigms with rather complicated setups, such as AAMT, to emphasize a user-friendly development of interventions and involve target groups to address their needs, e.g. by improving study support or providing more information material. We decided to address this issue in the upcoming RCT by providing more in-depth oral explanation by study therapists during the training phase.
One possible solution to this dilemma, as suggested by Beard and colleagues [70], is to increase credibility of the intervention by ensuring highly comprehensible and convincing treatment rationale and motivation.
Nevertheless, we took this as an indication to expand our study instructions and elaborate on the study rationale for the subsequent RCT. This approach may also be useful to increase the face validity and thus potentially the effectivity [73] of other digital mental health interventions, such as structured cognitive restructuring exercises [22,24], smartphone-based AAMT used for cognitive reappraisal [38–45], or original AAMT versions used to retrain approach and avoidance biases (see systematic review by Loijen and colleagues [74]).
One implication for future interventions using anti-depressive kinesthesia to augment cognitive restructuring derived from this feedback is that such interventions should be therapist-supported, at least when they are first delivered. Such support can maximize the intervention’s effects by leveraging the therapeutic relationship, which is a crucial common factor of psychotherapy [73].
In summary, results of our and previous studies are promising with regard to the clinical potential of a structured cognitive restructuring exercise as well as the potential of augmenting cognitive restructuring with anti-depressive kinesthesia. These findings are particularly important because structured cognitive restructuring exercises, which also lead to emotional insight through

augmentation with anti-depressive kinesthesia, could easily be used in mobile interventions without direct human interaction. The types of kinesthesia that are particularly beneficial and the extent to which they augment effects of structured cognitive restructuring on depressed and positive mood as posited by the ICS theory of depression [25] and studies demonstrating effects of manipulating facial expressions on depressed mood [29,30,34,36], will be subject of our subsequent RCT.

JARS–**Quant** | Table 2

Reporting Standards for Studies With an Experimental Manipulation (In Addition to Material Presented in Table 1)

Method

Experimental Manipulations

- Provide details of the experimental manipulation(s) intended for each study condition, including comparison conditions, and how and when experimental manipulations were actually administered, including
  - content of the specific experimental manipulations (if experimental manipulation is part of a clinical trial, address JARS–Quant Table 2: Module C)
    - summary or paraphrasing of instructions, unless they are unusual or compose the experimental manipulation, in which case they may be presented verbatim
  - method of experimental manipulation delivery
    - description of apparatus and materials used and their function in the experiment
      - specialized equipment by model and supplier
  - deliverer: who delivered the experimental manipulations
    - level of professional training
    - level of training in specific experimental manipulations
  - number of deliverers, and in the case of experimental manipulations, the *M*, *SD*, and range of number of individuals–units treated by each
  - setting: where the manipulations or experimental manipulations occurred
  - exposure quantity and duration: how many sessions, episodes, or events were intended to be delivered and how long they were intended to last
  - time span: how long it took to deliver the experimental manipulation to each unit
  - activities to increase compliance or adherence (e.g., incentives)
  - use of language other than English and the translation method
  - sufficient detail to allow for replication, including reference to or a copy of the manual of procedures; if the manual of procedures is available, describe how others may obtain it

During the training, participants completed four phases (see Figure 1): (1) the preparatory phase, (2) the depressed mood induction phase, (3) the first training phase, and (4) the second training phase. During the preparatory phase, participants were shown a fixation cross (3 sec) followed by different stimuli (8 sec) in randomized order on the smartphone. The stimuli consisted of self-recordings of the participants, words (in-)congruent with depressed mood (i.e., “hopeless” and “happy”), and words representing the so-called “cognitive triad” of depression (i.e., “me”, “the world”, and “the future”; [55]), which were each shown four times, resulting in 24 stimuli in total. Subsequently, in order to allow working with current depressed mood during the training, participants received a validated mood induction beforehand. During this mood induction phase, participants read aloud ten depressogenic cognitions shown on the smartphone screen (e.g., “My future is absolutely hopeless”). In addition, sadness-inducing music was played (an excerpt from “Adagio in G minor” by Tomaso Giovanni Albinoni). The induction procedure was previously validated [56,57].

Both training phases started with the study therapist providing instructions on how to engage in the trainings, followed by on-screen instructions with text and animations in the EmpkinS-EKSpression app. Then, participants completed two practice trials before starting the actual training. In the actual training, participants were confronted with 20 depressogenic (e.g., “I am a complete failure”; first training phase) and anti-depressive (e.g., “I can do this”; second training phase) statements presented in randomized order on the smartphone screen. The statements were derived from validated questionnaires in the context of depression (Beck Hopelessness Scale, German version [58]; Cognitive Triad Inventory, German version [59]; Automatic Thoughts Questionnaire, German version [60]; and Rosenberg Self Esteem Scale, German version [61]). A team of three experts in clinical psychology adapted the items to customize them for the intervention.

In the first training phase, participants were asked to *reject* the *depressogenic statements* in the following three invalidation steps: (1) reject the presented cognition, (2) affirm the rejection, and (3) self-support themselves. In each step, participants were able to choose from three statements (i.e., three statements expressing rejection, affirmation, and self-support, respectively) which they had to manually select on the screen and also *read aloud*. Their verbalization was to be amplified by the explicit expression of congruent kinesthesia, including mimic, gesture, and posture. Thus, their rejection of each depressogenic statement was performed with an expression of disapproval, the affirmation with an expression of approval, and self-support with an expression of joy (trials 1-10) and pride (trials 11-20).

In the second training phase, participants received instructions to *validate* the *anti-depressive statements* in the following sequence: (1) approve the positive cognition with an expression of approval, (2) affirm the approval with an expression of confidence, and (3) reinforce themselves

Units of Delivery and Analysis

- State the unit of delivery (how participants were grouped during delivery).
- Describe the smallest unit that was analyzed (and in the case of experiments, that was randomly assigned to conditions) to assess experimental manipulation effects (e.g., individuals, work groups, classes).
- Describe the analytical method used to account for this (e.g., adjusting the standard error estimates by the design effect or using multilevel analysis) if the unit of analysis differed from the unit of deliver.

with an expression of joy (trials 1-10) and pride (trials 11-20). Here, to conclude, participants read aloud all three statements they had selected.
After each trial, reinforcing statements and pictures were displayed on the screen. Throughout the sequence of training trials, study therapists rated the persuasiveness of participants' emotion expression, provided feedback to participants, and maintained control of the training app using a tablet.

Units of delivery are individual participants, as outlined in the "Participants" and "Procedures" sections.

Results

Participant Flow

- Report the total number of groups (if experimental manipulation was administered at the group level) and the number of participants assigned to each group, including
  - number of participants approached for inclusion
  - number of participants who began the experiment
  - number of participants who did not complete the experiment or crossed over to other conditions, with reasons
  - number of participants included in primary analyses
- Include a figure describing the flow of participants through each stage of the study (see JARS–Quant Participant Flowchart).

There was only one group involved in the study. The participant flow throughout the study is displayed in Figure 2.

Treatment Fidelity

- Provide evidence on whether the experimental manipulation was implemented as intended.

As can be seen in Figure 2, all participants completed the training.
Regarding safety and compliance, none of the participants terminated the training prematurely

Treatment Fidelity

- Describe baseline demographic and clinical characteristics of each group.

Baseline data is displayed in Table 1.

Adverse Events and Side Effects

- Report all important adverse events or side effects in each experimental condition. If none, state so.

To determine the safety of the intervention, study therapists recorded whether participants experienced significant mood deterioration during the training. The intervention was deemed safe if no more than 10% of the training sessions were prematurely terminated due to significant mood deterioration.
Regarding safety and compliance, none of the participants terminated the training prematurely

Discussion

- Discuss results, taking into account the mechanism by which the experimental manipulation was intended to work (causal pathways) or alternative mechanisms.
- Discuss the success of, and barriers to, implementing the experimental manipulation; fidelity of implementation if an experimental manipulation is involved.
- Discuss generalizability (external validity and construct validity) of the findings, taking into account
  - characteristics of the experimental manipulation
  - how and what outcomes were measured
  - length of follow-up
  - incentives
  - compliance rates

Describe the theoretical or practical significance of outcomes and the basis for these interpretations.

See “Discussion” section in the manuscript
