# Supplementary material for: The EmpkinS-EKSpression Reappraisal Training Augmented With Kinesthesia in Depression: One-Armed Feasibility Study
Source: JMIR Form Res. 2025 Apr 14;9:e65357. doi: 10.2196/65357 (PMC12038297; doi:10.2196/65357)
Supplement: Multimedia Appendix 2 [file formative_v9i1e65357_app2.docx]

How understandable did you find the instructions in the app?

| Not at all | A little | Fairly | Very |
| --- | --- | --- | --- |
| 🔿 | 🔿 | 🔿 | 🔿 |

How helpful did you find…

|  | Not at all | A little | Fairly | Very |
| --- | --- | --- | --- | --- |
| …the training with negative thoughts | 🔿 | 🔿 | 🔿 | 🔿 |
| …the training with positive thoughts | 🔿 | 🔿 | 🔿 | 🔿 |
| …the training as a whole | 🔿 | 🔿 | 🔿 | 🔿 |

Did you enjoy the training?

| 🔿 | Not at all |
| --- | --- |
| 🔿 | A little |
| 🔿 | Fairly |
| 🔿 | Very |

Would you continue the training regularly (e.g., 1x/week) if you had the opportunity to do so?

| 🔿 | Not at all |
| --- | --- |
| 🔿 | A little |
| 🔿 | Fairly |
| 🔿 | Very |

Has the training helped you to change your negative thoughts?

| 🔿 | Not at all |
| --- | --- |
| 🔿 | A little |
| 🔿 | Fairly |
| 🔿 | Very |

Has the training helped you to cope with your negative thoughts more appropriately?

| 🔿 | Not at all |
| --- | --- |
| 🔿 | A little |
| 🔿 | Fairly |
| 🔿 | Very |

How strenuous did you find the training?

| 🔿 | Not at all |
| --- | --- |
| 🔿 | A little |
| 🔿 | Fairly |
| 🔿 | Very |

Did you find the time required for the training appropriate?

| 🔿 | Far too high |
| --- | --- |
| 🔿 | A little too high |
| 🔿 | Appropriate |
| 🔿 | A little too short |
| 🔿 | Far too short |

Did you find it difficult to concentrate on the training?

| 🔿 | Not at all |
| --- | --- |
| 🔿 | A little |
| 🔿 | Fairly |
| 🔿 | Very |

How stressful did you find participating in the study?

| 🔿 | Not at all |
| --- | --- |
| 🔿 | A little |
| 🔿 | Fairly |
| 🔿 | Very |

How satisfied were you with the training in general?

| 🔿 | Not at all |
| --- | --- |
| 🔿 | A little |
| 🔿 | Fairly |
| 🔿 | Very |

Why did you take part in the study?

| 🔿 | Financial incentive |
| --- | --- |
| 🔿 | Pastime |
| 🔿 | Interest |
| 🔿 | Hoping for positive effects of the intervention |
| 🔿 | Other: ________________________ |

Would you recommend the training to friends or acquaintances?

| 🔿 | Yes |
| --- | --- |
| 🔿 | No |

What did you like less?

What would you change/add in the training?

What did you like most about the training?

What do you take away from the training?
